# Sternal wound tuberculosis following cardiac operations: a review

**DOI:** 10.5935/1678-9741.20140102

**Published:** 2015

**Authors:** Shi-Min Yuan

**Affiliations:** 1 The First Hospital of Putian, Teaching Hospital, Fujian Medical University, Putian, China.

**Keywords:** Sternum, Surgical Wound Infection, Tuberculosis

## Abstract

**Objective:**

The diagnosis and treatment of sternal wound infections with mycobacteria are challenging. Such an infection is often associated with a delayed diagnosis and improper treatment that may lead to a worsened clinical outcome. The present study is designed to highlight its clinical features so as to facilitate a prompt diagnosis and timely treatment.

**Methods:**

MEDLINE, Highwire Press, and Google search engine were searched for publications in the English language, with no time limit, reporting on sternal wound infection caused by tuberculosis after cardiac surgery.

**Results:**

A total of 12 articles reporting on 14 patients were included in this study. Coronary artery bypass grafting was the underlying surgical procedure in more than half of the cases. Purulent discharge and cold abscess were the two main presenting symptoms. Diagnosis of sternal wound infection was evidenced in all 14 patients by different investigations, with culture of samples being the most sensitive method of identifying the pathogen. Good response to first-line anti-tuberculous agents was noted. Almost all patients required surgical debridement/resection and, sometimes, sternal reconstruction. A delayed diagnosis of sternal wound infection may lead to repeated recurrences. A comparison between patients with sternal wound infection due to tuberculosis and non-tuberculous mycobacterial infections showed that the former infections took an even longer period of time. Comparisons also revealed patients with sternal tuberculosis infection had a significantly higher mortality than patients with sternal non-tuberculous infection (29.2% *vs*. 0%, *P*=0.051).

**Conclusion:**

Sternal infection caused by tuberculosis after cardiac surgery has a longer latency, better response to first-line drugs, and better outcomes in comparison with non-tuberculous sternal infection. Early diagnosis and early anti-tuberculous treatment can surely improve the patients' prognosis.

## Abstract (pt)

**Objetivo:**

O diagnóstico e tratamento da infecção esternal com micobactérias são desafiadores. Essa infecção é muitas vezes associada a um diagnóstico tardio e o tratamento inadequado que pode levar a um resultado clínico pior. O presente estudo tem como objetivo destacar suas características clínicas, a fim de facilitar um diagnóstico rápido e tratamento adequado em tempo hábil.

**Métodos:**

MEDLINE, Highwire Press, e o mecanismo de busca Google foram pesquisados por publicações em Inglês, sem limite de tempo, relatando sobre infecção de ferida esternal causada por tuberculose após cirurgia cardíaca.

**Resultados:**

Um total de 12 artigos, descrevendo 14 pacientes, foram incluídos no estudo. A revascularização do miocárdio foi o procedimento cirúrgico subjacente em mais da metade dos casos. Secreção purulenta e abscesso frio foram os dois principais sintomas apresentados. O diagnóstico de infecção de ferida esternal foi evidenciado em todos os 14 pacientes por diferentes investigações, com a cultura de amostras sendo o método mais sensível para identificar o agente patogênico. Boa resposta a agentes antituberculosos de primeira linha foi observada. Quase todos os pacientes necessitaram de desbridamento cirúrgico/ressecção e, em alguns casos, reconstrução esternal. Um diagnóstico tardio da infecção de ferida esternal pode levar a recorrências repetidas. Uma comparação entre pacientes com infecção de ferida esternal devido a infecções tuberculosas e micobactérias não tuberculosas mostrou que as infecções por tuberculose duraram um período de tempo maior. Comparações também revelaram que pacientes com infecção esternal por tuberculose tiveram mortalidade significativamente mais elevada do que os pacientes com infecção esternal por micobactérias não tuberculosas (29,2% *vs*. 0%; *P*=0,051).

**Conclusão:**

A infecção esternal por tuberculose após cirurgia cardíaca tem uma latência maior, melhor resposta aos medicamentos de primeira linha e melhores resultados em comparação com a infecção esternal por micobactérias não tuberculosas. O diagnóstico precoce e o rápido tratamento antituberculose podem certamente melhorar o prognóstico dos pacientes.

## INTRODUCTION

Mediastinitis is a serious complication of median sternotomy and is associated with significant morbidity and mortality^[1]^. Although sternal wound infections after cardiac operations through median sternotomy are uncommon, with a prevalence of only 0.4-5.0% of the cases^[2]^, they are associated with increased morbidity, prolonged hospital stay, and increased costs^[3]^. The risk factors of sternal wound infections have been sufficiently described^[4-7]^. The most common causative pathogen was *Staphylococcus aureus*, accounting for 28-58.1%, followed by *Acinetobacter spp* (20%)^[8-10]^.

Surgical wound infection caused by mycobacterium tuberculosis is extremely rare^[11]^. The exact prevalence remains uncertain; however, it has been estimated that sternal tuberculosis infection accounted for 4.1% of sternal wound infections after open heart surgery^[12]^. Recently, Unai et al.^[13]^ comprehensively studied the sternal wound infection caused by non-tuberculous mycobacteria, providing some detailed information on the patient. Nevertheless, there remains no clear consensus on sternal wound infection caused by tuberculosis after Wang et al.^[11]^ presented information of six patients. The diagnosis and treatment of sternal wound infections with mycobacteria are challenging. Such an infection is often associated with a delayed diagnosis and improper treatment that may lead to a worsened clinical outcome. Therefore, it is important for the physicians to bear in mind the clinical features of this rare infection. The aim of the present article is to make a comprehensive analysis of sternal wound infection caused by tuberculosis after cardiac surgery and compare it to the data available from the report by Unai et al.^[13]^ on sternal wound infections caused by non-tuberculous mycobacteria.

## METHODS

MEDLINE, Highwire Press, and Google search engine were searched for publications in the English language, with no time limit, reporting on sternal wound infection caused by tuberculosis after cardiac surgery. The terms "tuberculosis" and "coronary artery bypass", "heart valve replacement", "heart valve prosthesis", "heart valve repair", "sternotomy", "open heart surgery", and "cardiothoracic surgery" were employed for the searches. All the articles, titles, and subject headings were carefully screened for potential relevance. Sternal wound infections caused by non-tuberculous mycobacteria were excluded.

Due to the rarity of the condition, all the discovered articles reported only sporadic single or small series without a large population. Data were extracted mainly from the text. Variables included study population, demographics, clinical manifestations of sternal infection, predisposing risk factors, previous heart surgery, interval between cardiac surgery and sternal infection, sites of infections, diagnostic imaging, pathogen investigations, and anti-tuberculosis as well as surgical management strategies, length of follow-up, and main outcomes.

Numerical data were expressed as mean±SD and compared with the independent samples *t*-test. Count data were expressed as percentages and compared with the Fisher's exact test. Results with *P*<0.05 was considered statistically significant.

## RESULTS

Information on a total of 15 patients from 13 articles^[11,12,14-24]^ were collected. Data from repetitive descriptions of the same patient in 2 articles^[23,24]^ were incorporated. As a result, 12 articles reporting on 14 patients were included in this study. The 12 articles were comprised of 10 case reports^[11,14-21,23]^ and 2 original articles^[12,22]^. Gender of 11 patients was described, including 8 (72.7%) males and 3 (27.3%) females. The patients' age was 58.6±15.3 (range, 16-72; median, 60) years (*n*=11). The underlying surgical procedures were coronary artery bypass grafting in 8 patients (57.1%), open heart surgery (unspecified) in 3 patients (21.4%) as well as aortic valve replacement, mitral valve repair, redo-Bentall operation, and cardiothoracic surgery (unspecified) in 1 (7.1%) patient each, respectively. The interval between heart surgery and sternal infection was 13.3±17.1 (range, 0.5-60; median, 7) months (*n*=11). The symptoms were described in 12 patients and included purulent discharge in 7 (58.3%), cold abscess in 4 (33.3%), subcutaneous sinus in 3 (33.3%), local pain in 2 (16.7%), fever in 2 (16.7%), sternal swelling in 1 (8.3%), sternal mass in 1 (8.3%), and symptoms irrelevant to sternal wound infections in 2 (16.7%) patients. In 6 (42.9%) patients, one or more predictive risk factors for sternal wound infection were determined, which were diabetes mellitus in 4 (66.7%) (of those, one patient was also associated with hypertension, hyperlipidemia, and diabetic nephropathy requiring persisted dialysis, and another patient was associated with lung tuberculosis) and tuberculosis contact in 2 (33.3%) patients. The locations of infections in the sternum were described in 6 patients, including 1 (16.7%) in the manubrium^[16]^, 1 (16.7%) in the upper portion^[18]^, 2 (33.3%) in the lower portion^[11,15]^, 1 (16.7%) in the body of the sternum^[20]^, and 1 (16.7%) that was described as "9 cm below the suprasternal notch"^[14]^. Sternal destruction was noted on chest X-ray and on chest computed tomography in 2 (14.3%) patients each. Lymphadenopathy was noted in 3 (21.4%) patients: cervical^[16]^, hilar and subcarinal^[21]^, and scattered visceral lymphadenopathy^[23]^ in 1 patient each. Diagnosis of sternal wound infection was evidenced in all 14 patients by different investigations, with culture of samples being the most sensitive method for identifying the pathogen (Table 1).

**Table 1:** Investigations of samples for the diagnosis of sternal wound tuberculosis infection.

Investigation	Sample	*n* (%)	Reference
Cultures		12 (85.7)*	
	Intraoperative resected/debridged tissue	7 (58.3)	[11,14,15,18-21,23]
	Sternal pus/discharge	3 (25)	[17,18,22]
	Ascites	1 (8.3)	[23]
	Sputum	1 (8.3)	[17]
	Unspecified	2 (16.7)	[12]
Histopathology		9(64.3)	
	Intraoperative resected/debrided para-sternal tissue	4 (44.4)	[11,14,19,20]
	Fine needle aspiration of cervical lymph nodes	1 (111)	[16]
	Resected pulmonary lesion	1 (111)	[17]
	Intraoperative frozen nodule biopsy	1 (111)	[23]
	Unspecified specimens	2 (22.2)	[12]
Ziehl-Neelsen stain		3 (21.4)	
	Intraoperative resected/debridged specimens	2 (66.7)	[11,19]
	Sputum & pus	1 (33.3)	[17]
Polymerase chain reaction		1 (7.1)	
	Debrided tissue	1 (100)	[11]

* There were a total of 14 samples for mycobacterial cultures from 12 patients.

Associated *Staphylococcus aureus* infection was found in 2 patients^[11,15]^. Anti-tuberculous treatment was indicated in 12 patients. One of them received an adjusted anti-tuberculous regimen due to end-stage renal failure, gastrointestinal upset, and thrombocytopenia^[11]^. Duration of anti-tuberculous treatment was 10.8±1.6 (range, 9-12; median, 12) months (*n*=5). Anti-tuberculous therapy took effect within various time intervals, either rapidly^[23]^ or over a few weeks^[16]^. Discharge from the sinus stopped in 15 days and the sinus healed after 2 months^[14]^. Surgical operation was performed in 13 patients: debridement in 6 (46.2%)^[11,14,16,18,21,22]^, extensive resection with chest wall reconstruction in 5 (38.5%)^[15,19,20,23]^, and the surgical procedure was not indicated in 2 (15.4%) patients^[12]^. The chest reconstruction materials were pectoralis (major) flap in 3 (60%)^[15,19,20]^, omental flap interposition plus titanium plate in 1 (20%)^[23]^, and pectoralis major myocutaneous flap in stage 1 and omental flap in stage 2 operation in 1 (20%) patient^[23]^. The patients were at a follow-up of 9±3.9 (range, 3-14; median, 9) months (*n*=9). Prognosis was not reported in 2 patients^[19,22]^. All the remaining 12 patients survived. However, before a full recovery, 3 (25%) patients had 1-2 recurrences due to an up to 2-year delay in the diagnosis of tuberculous infection^[14,17,18]^.

## DISCUSSION

Dissemination of tuberculosis include spread as a late complication of pulmonary tuberculous, reactivation of latent foci formed during hematogenous or lymphatic dissemination of primary tuberculosis, or direct extension from mediastinal lymph nodes^[25]^. Skeletal tuberculosis accounted for approximately 6-10% of extrapulmonary tuberculous cases and 1% of all tuberculous cases, and sternal tuberculosis is involved in approximately 1% of skeletal tuberculosis cases^[26]^. Sternal infection due to tuberculosis after cardiac surgery is even rarer.

Mycobacterium tuberculosis is a member of the slow-growing pathogenic mycobacterial species, characterized by a 12- to 24-hour division rate and prolonged culture period on agar of up to 21 days^[27]^. Hosts of tuberculous infections may be in a latent period with no symptoms for years or decades, allowing the establishment of a chronic asymptomatic infection, followed by reactivation and transmission years later to new uninfected hosts^[27]^.

This study showed that 8 (57.1%) patients with sternal wound infection caused by tuberculosis had a history of coronary artery bypass grafting. The most common manifestation was purulent discharge, followed by cold abscess. The diagnosis of sternal infection due to tuberculosis can be made primarily from the bony destruction, and eventually it will depend on pathogen investigations by culture and histopathology of aspirated/debrided/resected tissue. Besides, Ziehl-Neelsen stain and polymerase chain reaction can be valuable for pathogen screening. Delayed diagnoses may lead to recurrence and protracted course of disease. All patients responded well to first-line anti-tuberculous drugs. Most of the patients required surgical treatment, with nearly half requiring extensive resection with chest wall reconstruction.

Rapidly growing mycobacteria is largely present in our living environment. It is usually resistant to first-line anti-tuberculosis agents^[28]^ in addition to being commonly resistant to sterilizers, disinfectants, and antiseptics^[29]^. Therefore, non-tuberculous mycobacteria may contaminate medical devices such as heart valve prosthesis, and it can be associated with nosocomial outbreaks.

The average time from the operation to sternal non-tuberculous mycobacterial infection was 64.1±84.6 (range, 24-330; median, 30) days^[13]^, which seems to be longer than the latency of 1-2 months of usual bacterial mediastinitis^[3]^. The present study demonstrated that the sternal infections caused by tuberculosis required an even longer time to develop than sternal infections with non-tuberculous mycobacteria. Comparisons also revealed patients with sternal infection caused by non-tuberculous mycobacteria had significantly higher mortality rates than patients with sternal infection due to tuberculosis (29.2% *vs*. 0%, *P*=0.051) (Table 2).

**Table 2:** A comparison between tuberculous and non-tuberculous mycobacterial stemal infections.

Variable	TB	NTB	*P* value
Patients’ age	58.6±15.3	55.3±16.6	0.619
Gender (male/female)	8/3	43/16	0.521
Latency from cardiac surgery to sternal infection (month)	13.3±17.1	1.4±1.1	0.044
Purulent discharge	7/13	27/38	0.315
Mortality	0/12	14/48	0.051

NTB=non-tuberculosis; TB=tuberculosis

In general, sternal infection caused by tuberculosis after cardiac surgery has longer latency, better response to first-line drugs, and better outcomes in comparison with sternal infection caused by non-tuberculous mycobacteria. Early diagnosis and early anti-tuberculous treatment can surely improve the patients' prognosis.

**Table:** 

**Authors’ roles & responsibilities**	
SMY	Study conception and design; analysis and/or interpretation of data; manuscript writing.
